# Randomised controlled trials of nursing interventions in spinal cord injury: a scoping review

**DOI:** 10.1038/s41394-026-00749-x

**Published:** 2026-09-03

**Authors:** Louise C. Kelly, Joanne V. Glinsky, Lisa A. Harvey

**Affiliations:** 1 The John Walsh Centre for Rehabilitation Research, Faculty of Medicine and Health, University of Sydney, Sydney, NSW Australia; 2 Sydney Local Health District, Faculty of Medicine and Health, University of Sydney, Sydney, NSW Australia

**Keywords:** Health care, Health occupations

## Abstract

**Study design:**

Scoping Review

**Objectives:**

Randomised controlled trials (RCTs) are considered the gold standard for evaluating the effectiveness of clinical interventions. In spinal cord injury (SCI) care, nurses deliver many interventions, yet the number, quality, and level of nurse involvement in RCTs remains unclear. Poorly designed RCTs can introduce bias, and limited nurse participation may restrict the development of evidence-based nursing practice. This review aimed to determine: 1. The number and topics of RCTs on nurse-related interventions for people with SCI; 2. The extent of nurse leadership and participation in these RCTs; 3. The methodological quality of the RCTs.

**Setting:**

Australia

**Methods:**

Three databases were searched over a 20-year period for RCTs involving people with SCI and interventions relevant to nursing practice. Two authors independently screened studies for inclusion. Extracted data included study location, sample size, intervention type, nurse involvement, and authorship by nurses. Methodological quality was assessed using the PEDro scale. The protocol was registered with Open Science Framework (https://osf.io/k9xde), and PRISMA-ScR guidelines informed reporting.

**Results:**

Forty-six RCTs were included from 29,744 retrieved articles. Five main intervention categories were identified, with most studies focusing on bladder and wound management. Studies were primarily conducted in Europe, Asia, and North America. Sample sizes ranged from 9 to 489 participants, with a median (IQR) of 61 (37–91). Nurses were authors on 29 out of the 46 trials, and 26% were nurse led. PEDro scores ranged from 2 to 9, with a median (IQR) of 5 (5–6.8).

**Conclusions:**

Few RCTs have evaluated nursing interventions for people with SCI. Many studies showed susceptibility to selection bias. Greater nurse leadership and participation in high-quality RCTs are needed to strengthen evidence-based SCI nursing care.

## Introduction

Randomised controlled trials (RCTs) are considered the gold standard for determining whether a clinical intervention is effective. This is because they minimise bias [1]. Nurses in SCI settings deliver a broad range of complex interventions to stabilise patients and support recovery [2] but it is not clear how many relevant RCTs exist to support nursing practice in SCI care. This type of information is important as a first step to better understanding the evidence supporting the various interventions nurses administer as it has been reported that up to 40% of patient care is not supported by the best available evidence [3]. Therefore, the first purpose of this scoping review was to explore how many RCTs involving nursing interventions for people with SCI have been conducted and in what areas.

Nursing research is critical not only for improving practice and outcomes, but also for elevating the unique contributions of nursing within multidisciplinary teams. It allows nurses to generate context-specific, evidence-based knowledge that reflects their roles and responsibilities [4]. SCI Nurses are well positioned to contribute to RCTs as they are most likely to be administering and monitoring the response of patients to interventions. They therefore have an in depth understanding of the key questions that can or should be investigated. The involvement of nurses in RCTs is also important to the development of the nursing profession. It helps nurses develop skills in research as well as clinical decision making. This ultimately leads to improved patient care [5]. Yet no-one has explored how involved nurses are in the leadership or conduct of RCTs. Therefore, a secondary purpose of this scoping review was to determine how many RCTs were nurse initiated/led or included nurses in the research team.

Whilst RCTs are considered the gold standard they too are vulnerable to bias if poorly conducted. Poor-quality RCTs present substantial threats to the credibility of clinical research and the advancement of evidence-based practice. The risk is that bias compromises the internal validity of any findings [6]. Dissemination of unreliable evidence can distort future systematic reviews, misguide clinical practice guidelines and ultimately undermine patient care [7]. For this reason, as part of this scoping review, we looked at the methodological rigor of the RCTS we identified. We used the PEDro score to measure this.

## Methods

This scoping review is reported in accordance with the guidance provided by the Preferred Reporting items for Systematic Reviews and Meta-Analyses extension for Scoping Reviews (PRISMA-ScR) checklist [8]. This checklist is provided in supplementary file 1.

### Eligibility criteria

Studies were included in this scoping review if:

- they were RCTs
- 75% of participants had an acquired traumatic or non-traumatic SCI
- they were published in English
- they occurred in any clinical or home setting
- the intervention being studied would typically be provided by a nurse (Registered Nurse, Nurse Specialist, Nurse Consultant, Nurse Practitioner, Nurse Educator, Nurse Manager, Advanced Practice Nurse)
- They examined the effectiveness of nursing intervention used for or as part of:
  - Bladder management
  - Bowel management
  - Skin management
  - Pressure injury (PI) / Wound management
  - Health Literacy interventions
  - Telehealth
  - Sexuality/Fertility interventions
  - Case Management
  - Pain management interventions
  - Peer support programs
  - Programs using psychological methodology (eg. motivational interviewing, health coaching etc.
  - Complimentary medicine
  - Ventilation management
  RCTs were excluded if they examined:
  - Surgery
  - Functional training interventions
  - Exercise or sport activities
  - Psychological therapy
  - Dietetics
  - Spasticity treatments
  - Respiratory therapies

### Search strategy

Three main databases (MEDLINE, Embase and CENTRAL) were searched via OVID® in March 2025. Studies were included if they were published between 1 January 2004 and 31 December 2024. The comprehensive search strategy built upon pre-existing search strategies for SCI [9, 10] as well as RCTs [10–12]. The search was adjusted for each database and can be viewed in supplementary file 2. A preliminary search was conducted to ensure the search terms were capturing relevant studies. In addition, a medical/healthcare librarian with experience in systematic reviews was consulted to refine the search terms prior to its finalisation [13]. The references of any identified papers were also screened.

### Search outcome

The final searches were executed on 25th March 2025. Results for the searches were imported as separate libraries into EndNote x20 [14] and duplicates were removed prior to merging into one library. Two levels of screening were undertaken including an initial screen on the basis of the title and abstract, followed by a full screen on the basis of the full publication. This screening process was focused on the stated inclusion criteria. At both levels of screening, search results were independently reviewed by two authors with a plan to use the third author if consensus could not be reached however, adjudication was not required.

### Data charting

The final list of RCT papers were then reviewed to extract specific data. These data included: the journal reference, the discipline of the first and last author, whether any author had a nursing background, whether any nurses participated in the trial in any capacity (eg. recruiting participants, delivering the intervention, collecting data etc), numbers of participants, location of the trial, details to enable methodological assessment and the category of intervention. The data were captured on a data chart which was developed by the first author (LK) and pilot tested and reviewed by the two other authors (LH & JG). Data were extracted independently and cross referenced against the original article by a second author to ensure accuracy of the extracted information.

### Methodological assessment

The included RCTs were assessed for methodological quality using the PEDro scale. The PEDro scale focuses on the internal validity and statistical reporting. It is a validated tool that has high inter-rater reliability and is simple to use [15]. The PEDro includes 11 items but only 10 are summed up for a total score. A higher score reflects a better quality trial with less susceptibility to bias than a lower score [16]. LK and LH independently rated the RCTs with the third author arbitrating any differences.

### Synthesis

Results were grouped and reported according to the type of intervention. A narrative synthesis outlines the number of RCTs, authors’ disciplines and PEDro scores in the results section.

## Results

**Fig. 1:**
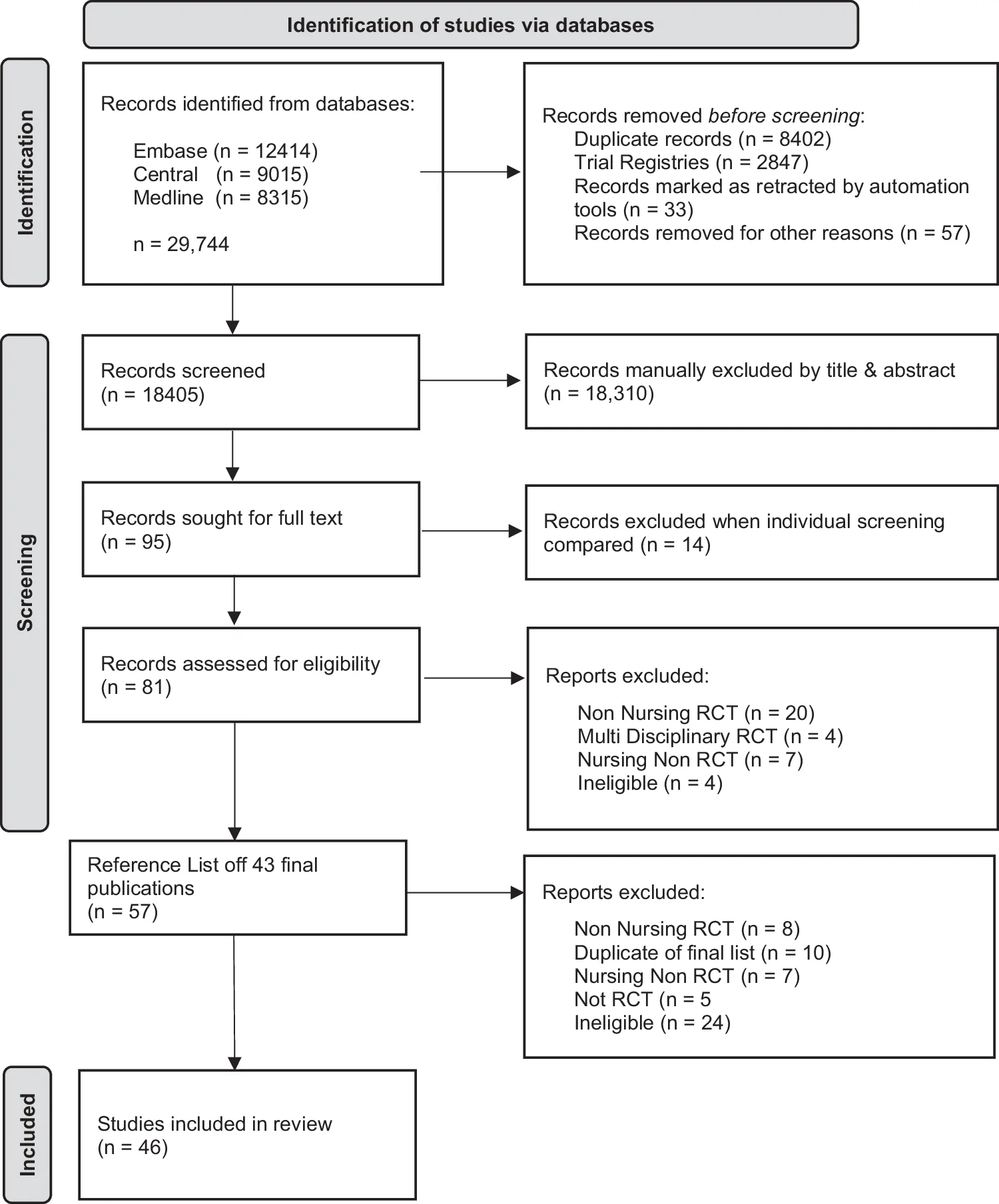
The PRISMA Flow Diagram detailed the number of papers screened and ultimately included in this scoping review.

The searches retrieved 29,744 potential articles. Of these 11,339 were duplicates or articles that did not meet the inclusion / exclusion criteria. A further 18,310 articles were excluded on title/abstract and a final 36 were excluded on full text. The reference list of the remaining 43 publications was screened to provide a final total of 46 RCTs which were included in this review (Table 1). Figure 1 displays the Prisma flow diagram.

**Table 1:** Summary table of included RCTS.

Paper	1st Author Nurse	Any Author Nurse	Nurse used in trial	Category of Intervention	Sample size (n=)	Location	Setting	Intervention	Control		Pedro Score
Arora, Harvey et al. [30]	No	Yes	Yes^a^	Wound Management	120	Bangladesh	Community	Weekly phone call for advice for 12 weeks	Usual care		8
Asgari, Zolfaghari et al. [31]	Yes	Yes	Yes ^a,b,c^	Wound management	70	Iran	Inpatient	Silver nanoparticle dressings	hydrocolloid dressings		6
Biering-Sørensen, Hansen et al. [32]	No	No	Yes ^c^	Bladder Management	24	Denmark	Community	Compact intermittent catheter	Standard length intermittent catheters		3
Bonfill, Rigau et al. [33]	No	No	Yes^a^	Bladder Management	489	Spain, Portugal, Chile, Turkey, Italy	Inpatient	Silver alloy coated catheters	All other catheters		9
Cardenas, Hoffman et al. [34]	No	Yes	Yes ^a,c^	Bladder Management	56	United States of America	Community	Bladder education program and review/optimisation of bladder management	Usual methods of bladder management		4
Cardenas and Hoffman [35]	No	No	Yes^d^	Bladder management	56	United States of America	Community	Hydrophilic catheters	PVC nelaton catheters		4
Cardenas, Moore et al. [36]	No	Yes	Yes^a,c^	Bladder management	224	United States of America	Inpatient / Community	Hydrophilic catheters	polyvinyl chloride (PVC) catheters		6
Carlson, Vigen et al. [37]	No	Yes	Yes^c^	Health Literacy	166	United States of America	Community	In home and telehealth lifestyle based support PI prevention	Brochure – PI prevention		7
Chartier-Kastler, Lauge et al. [38]	No	No	Yes^d^	Bladder management	36	France, Denmark	Community	Compact intermittent catheter	standard length intermittent catheter		5
Christensen, Bazzocchi et al. [39]	No	Yes	Yes^c^	Bowel Management	87	Sweden, Italy, Germany, England, Denmark	Inpatients	Trans anal irrigation	Standard care		7
Coggrave and Norton [40]	Yes	Yes	Yes^a,b,c,^	Bowel Management	68	England	Community	Stepwise bowel protocol	Usual care		6
Costa, Menier et al. [41]	No	No	No	Bladder Management	81	United States of America	Community	30 cm intermittent catheter	40 cm intermittent catheter		5
De Ridder, Everaert et al. [42]	No	No	No	Bladder Management	123	Belgium, Spain	Inpatients	Hydrophilic catheters	polyvinyl chloride (PVC) catheters		5
Domurath, Kutzenberger et al. [43]	No	No	Yes^c^	Bladder Management	37	Germany	Community	Telescoping intermittent catheter	Standard length intermittent catheter		7
Dwivedi, Srivastava et al. [17]	No	No	No^f^	Wound Management	60	India	Inpatients	Negative pressure wound therapy	Usual wound care		4
Evans, Hill et al. [44]	No	Yes	Yes ^a,e^	Health Literacy	61	United States of America	Residential, Community and Inpatients	Nurse administered education intervention	Usual care		5
Guihan, Bombardier et al. [45]	No	Yes	Yes^a,b,c,e^	Telehealth/Health Literacy	143	United States of America	Community	Telephone based individual motivational interview counselling plus a self management skills group	Telephone based individual education counselling plus group education		6
Ho, Bensitel et al. [46]	No	No	Yes^a,c^	Wound Management	60	United States of America	Inpatients	Low – pressure Pulsatile lavage	sham		7
Hollisaz, Khedmat et al. [47]	No	No	Yes^a^	Wound Management	91	Iran	Inpatients	Hydrocolloid	Phenytoin cream	Usual care	7
Hossain, Harvey et al. [48]	No	No	No	Telehealth – Transitional Support	30	Bangladesh	Community	Regular phone contact and 3 home visits	Telephone call and option for home visit		8
Hossain, Harvey et al. [49]	No	No	Yes^a^	Telehealth – Transitional Support	410	Bangladesh	Community	36 phone follow up and three home visits on top of usual care	Usual care		6
Houlihan, Jette et al. [50]	No	No	Yes^d,e^	Telehealth – Health Literacy	142	United States of America	Community	Telehealth intervention via “CareCall” PI, depression, health care utilisation	Usual care		7
Huang, Pacheco Barzallo et al. [51]	Not able to determine	Not able to determine	Yes^d,e^	Bladder management	80	China	Inpatients	Quality Control Circle	Health education for Intermittent Catheter use		5
Irgens, Midelfart-Hoff et al. [52]	No	No	Yes^a^	Wound Management	56	Norway	Community	Virtual wound clinic	Regular outpatient care		5
Kaya, Turani et al. [53]	No	No	No^f^	Wound Management	49	Turkey	Inpatients	Hydrogel	Povidone-iodine gauze		4
Kelly, Glinsky et al. [54]	Yes	Yes	Yes^a,b,c^	Bowel Management	20	Australia	Inpatients	Microenema delivered via 10 mL syringe with 10 cm nelaton catheter	Standard micro enema		9
Kim and Cho [55]	Yes	Yes	Yes^a,d,e^	Health Literacy	47	Korea	Inpatients	Self efficacy enhancement program for pressure injury prevention	Pressure injury prevention brochure		5
Kryger, et al. [56]	No	No	Yes^a,c^	Telehealth – Health Literacy	38	United States of America	Community	Supporting self management via (iMHere) ap, prompts, record data, advice	Usual care (in clinic appointments and access to nurse for recommendations/triage clinic review)		5
Kumar, Akriti et al. [57]	No	No	No^f^	Wound Management	22	India	Inpatients	Eusol solution	Silverstream solution		2
Li, Li et al. [58]	Yes	No	Yes^a,b,c,e^	Telehealth – Transitional support	78	China	Community	‘Wechat” platform	Regular care		5
Li, Li et al. [59]	Yes	Yes	Yes^a,b,c^	Telehealth – Transitional support	80	China	Community	Online home nursing care	Routine discharge guidance		5
Liu, Deegan et al. [60]	Yes	No	Yes^a,e^	Telehealth – Health Literacy	39	England	Community	Text messaging	Usual care		4
Moore, Burt et al. [61]	Yes	No	Yes^a,b,d^	Bladder Management	36	Canada	Inpatient	Clean intermittent nurse catheterisation	Sterile intermittent nurse catheterisation		6
Pain, Soopramanien et al. [62]	No	No	Yes^a^	Telehealth – Transitional support	137	England, Italy and Belgium	Community	Standard post discharge support plus videoconferencing	Standard post discharge support		3
Rintala, Garber et al. [63]	No	No	Yes^a,e^	Health Literacy	49	United States of America	Community	Individualised Pressure Injury education structured phone follow up monthly	Monthly mail or telephone follow up without educational content	Quarterly mail or telephone follow up without educational content	6
Ruijuan, Li et al. [64]	Yes	Yes	Yes^a^	Bladder management	92	China	Community	Catheter follow up management	Routine treatment		5
Sarica, Akkoc et al. [65]	No	No	Yes^d^	Bladder management	25	Turkey	Inpatients	Hydrophilic catheter	Gel lubricated catheter	Poly vinyl chloride (PVC) catheter	5
Scheel-Sailer, Koligi et al. [66]	No	No	Yes^a,e^	Wound Management	30	Switzerland	Inpatients	Computerised decision support system	Standard care		5
Sedghi Goyaghaj, Pishgooie et al. [67]	Yes	Yes	Yes ^a,b,c,e^	Health Literacy	60	Iran	Community	Self care program training	Usual care		5
Subbanna, Margaret Shanti et al. [68]	No	No	Yes^a^	Wound Management	28	India	Inpatients	Phenytoin solution	Normal saline		9
Sundby, Irgens et al. [69]	No	No	Yes ^a,c^	Wound Management	9	Norway	Community	Intermittent negative pressure plus standard wound care	Standard wound care		5
Taly, Krishan et al. [70]	No	No	Yes ^a,c^	Wound Management	35	India	Inpatients	Multiwave light therapy	sham		8
Waites, Canupp et al. [71]	No	Yes	Yes ^a,b,c,d^	Bladder Management	89	United States of America	Community	Normal saline	Acetic acid	Meomycin-polymyxin	6
Wang, Wang et al. [72]	Yes	Yes	Yes ^a,c,e^	Health Literacy	74	China	Community	Twice weekly follow for 1 month, once weekly follow up for 1 month, follow up at 6 months	Routine nursing care		5
Zhang, Xia et al. [73]	Yes	No	Yes^a^	Bowel Management	184	China	Inpatient	Quantitative assessment based nursing intervention	Routine care		5
Zhang, Cai et al. [74]	No	Yes	No	Bladder Management	87	China	Inpatient	Low frequency bladder vibration	Routine care		4

^a^Delivered intervention.

^b^Recruitment.

^c^Data collection/assessment.

^d^Provided instruction on intervention device.

^e^Design of intervention.

^f^It was not clearly reported whether the investigators or nursing staff completed the dressing changes.

**Fig. 2:**
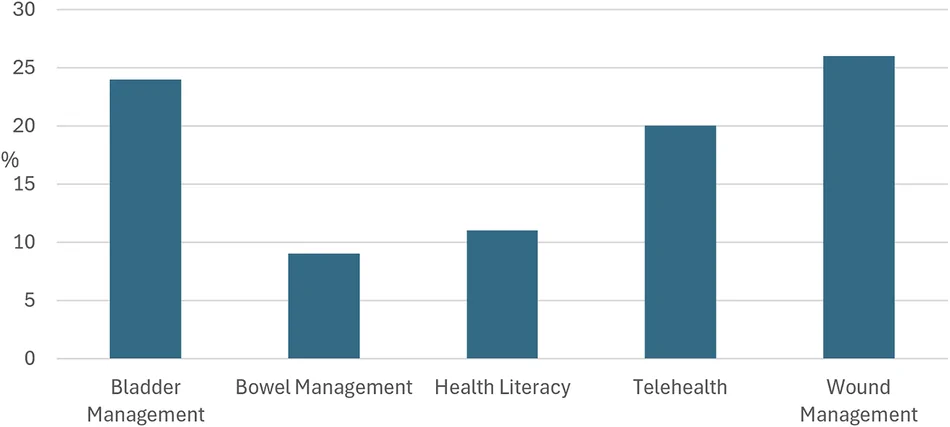
Percentage of trials by category.

There were five main categories of interventions relevant to nursing identified in this scoping review (Fig. 2). Most RCTs were conducted in the Northern Hemisphere, namely; Europe, Asia and North America. The others were conducted in Australia and Chile. The sample size of the RCTS varied greatly from 9 to 489 participants. The median (IQR) sample size was 61 (37 to 91). Twenty five RCTs were conducted in the community, 19 during hospitalisation and two RCTs crossed from inpatients to community.

### Nursing involvement in the identified randomised controlled trials

**Fig. 3:**
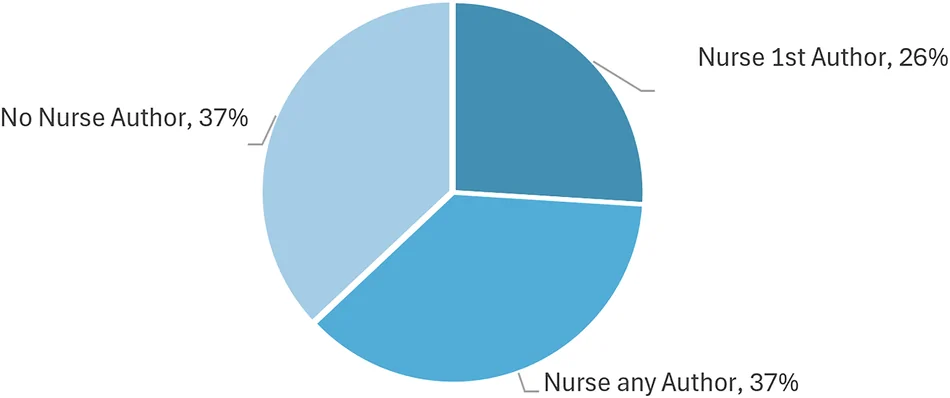
Percentage of nursing authorship.

**Fig. 4:**
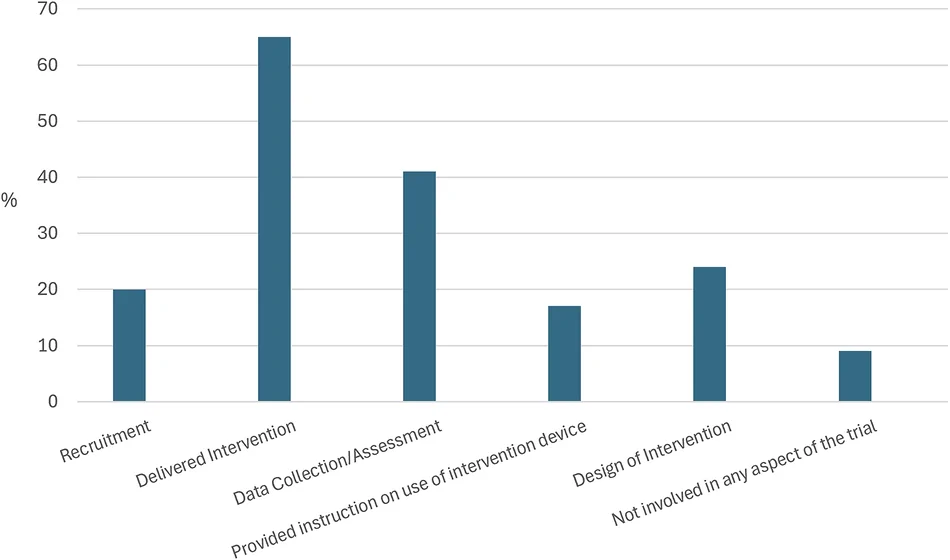
Percentage of nursing involvement in different aspects of the trial.

A nurse was identified as first author in 12 RCTs (26%) and any author in 17 RCTs (63%). Figure 3 provides a summary of nursing authorship. Nurses participated in 39 (85%) of the identified RCTs. This included delivering the intervention/control, educating participants on how to use the intervention, assessing or collecting outcome measures and recruitment of participants. A summary is provided in Fig. 4. In RCTs involving education, health literacy or decision-making programs, nurses provided clinical input into the design and development of the intervention. There were 3 wound management RCTs where it was unclear whether nurses were involved in the assisting with the wound management in the intervention or control groups as this was not reported. The exception was Dwivedi, Srivastava et al. [17] who reported that the resident staff or research team (medical officers) changed the negative pressure therapy dressing. Four of the 46 RCTs did not involve nurses in any part of the trial.

### Interventions for bladder management

The majority of RCTs in this review (n=15, 33%), related to the management of the neurogenic bladder. Nine RCTs focused on intermittent catheterisation (Table 2).

**Table 2:** Trials involving intermittent catheterisation.

coated vs non coated catheters	4
short vs standard length	4
sterile vs clean technique	1

Two RCTs involved focused training programs designed to improve the ability of people with SCI to understand, access and use health information to make informed decisions about their bladder management (intensive health literacy programs). One RCT compared a silver coated foley catheter to standard foley catheters. One RCT compared three different bladder instillation solutions to treat bacteria in the urine and one RCT compared bladder vibrations to standard care for prevention of urinary tract infection (UTI).

### Nursing involvement in bladder management RCTs

Nurses were in involved in 12 bladder management RCTs (80%). Despite all these RCTs using nurses to either provide the intervention or act as a blinded assessor, only 5 of the 15 RCTs (33%) included at least one nurse as an author and only 3 RCTs (20%) had a nurse as either first or last author (indicative that the trial was nurse led).

### Interventions for wound management

Twelve RCTs (26%) focused on wound management. The majority of RCTs compared one type of dressing product or solution to another (n = 7, 58%) One RCT included participants solely with leg ulcers and examined sequential compression therapy. The remaining RCTs dealt with pressure injuries (PIs) and examined a variety of interventions including negative pressure wound therapy, lavage and light therapy. One RCT from a low to middle income country (LMIC) examined PI advice provided by telephone. Another measured adherence to a specific treatment process for PI management using a computerised decision support system.

### Nursing involvement in wound management RCTs

All 12 RCTs relied upon nurses to either provide the intervention, or act as a blinded assessor and in one, a nurse recruited participants. The use of nurses was unclear in 3 RCTs. Nurses were authors on 3 RCTs (25%) and acknowledged in the manuscript in one other trial. Only one trial out of these three had a nurse as first and last author.

### Interventions for telehealth

Nine RCTs (18%) compared telehealth as a modality to deliver health interventions with usual care. Fifty five percent of the RCTs focused on clinical support or advice for people who had recently been discharged home following initial injury. The remaining 4 RCTs focused on improving health literacy on common complications of SCI such as preventing and managing PIs or improving self management strategies for their injuries.

### Nursing involvement in telehealth RCTs

Three RCTs were nurse led (33%). Nurses were involved in delivery of the intervention in 7 RCTs and acknowledged as an author in 2. Nurses were used to recruit participants in 3 RCTs, act as an assessor in four RCTs and provided clinical input into the development of the intervention in one RCT.

### Interventions for health literacy

Six RCTs related to improving the health literacy of people with SCI (13%).

Three of the RCTs investigated the effectiveness health literacy programs on PI prevention, and 1 RCT focused on self–efficacy / self management. One RCT focused upon improved health literacy around Methicillin Resistant Staphylococcus Aureus and one provided intensive rehabilitation (education, resource book and exercises) in the home post discharge.

### Nursing involvement in health literacy RCTs

Three of the health literacy RCTs were nurse led and 5 included nurses as an author. All six RCTs used nurses to deliver the intervention. In five of the RCTs, nurses provided clinical input into the details of the intervention and collected data in 3 RCTs. Nurses were involved in recruitment in one RCT and providing education of the intervention in one other.

### Interventions for bowel management

Four RCTs (9%) studied interventions for bowel management. Most of the RCTs were comparing previously accepted neurogenic bowel care interventions against usual care except for trans anal irrigation, which was a novel intervention for use in the SCI population at the time.

### Nursing involvement in bowel management RCTs

Seventy five percent of RCTs for bowel management were nurse led. Three RCTs acknowledged nurses as authors and 100% of the RCTs used nurses to deliver the intervention. Three RCTs utilised nurses for data collection and nurses were involved in recruiting participants in 2 RCTs.

### Methodological quality

**Fig. 5:**
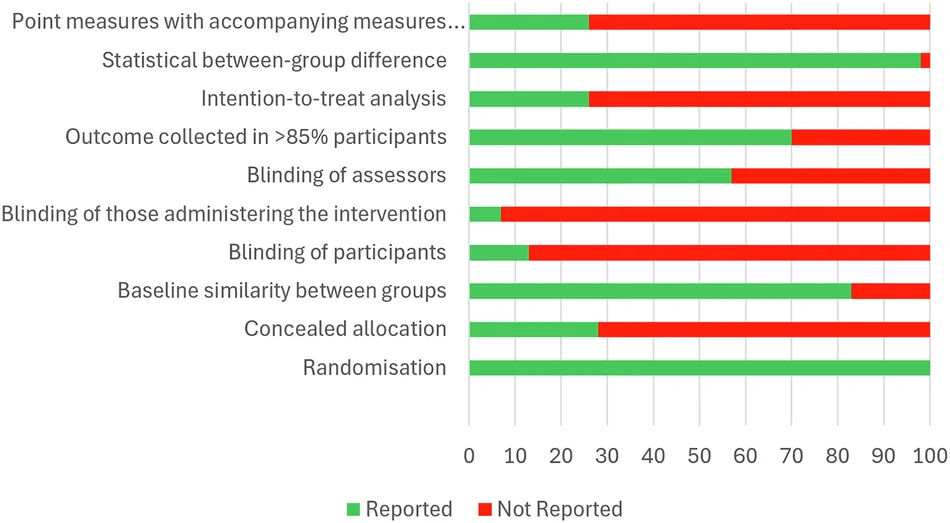
A summary of the PEDro scores as a percentage.

PEDro scores ranged from 2 to 9 with a median (IQR) of 5 (5 to 7) indicating that the RCTs were moderately vulnerable to bias and of fair methodological quality [18]. Figure 5 highlights a summary of the PEDro scores for all RCTs. The majority of RCTs were at risk of bias due to inadequate blinding, incomplete outcome data (fewer than 85% of participants assessed), and failure to use an intention-to-treat analysis. Additionally, only 18 RCTs reported using concealed allocation.

## Discussion

The aim of this scoping review was to identify how many RCTs had been conducted on a SCI nursing-related topic, the degree of involvement of nurses in the identified RCTs and the methodological quality of RCTs. We were surprised to only identify 46 RCTs published since 2004, many of which did not include nurses as authors and most of which had important methodological weaknesses.

This review reveals that in SCI nursing, the available research evidence is both limited in volume and variable in quality. Many interventions delivered daily by nurses remain underpinned by relatively few rigorous RCTs. This review also found that interventions for bladder management and wound care were investigated in more than half of the identified RCTs. This is not unexpected as urinary complications such as UTIs are the second most common cause of mortality in people with SCI [19] and the incidence of PI in this population is reported to be as high as 29%, globally [20]. Pressure injuries are also associated with high mortality rates. Randomised controlled trials relating to health literacy and bowel management were found to have greater nurse involvement. This no doubt reflects the pivotal role nurses play in educating people with SCI about the implications of their injuries on all aspect of their health [21]. Similarly, bowel care is a core nursing skill and critical in SCI where up to 60% of people with SCI are adversely affected by ongoing bowel dysfunction [22].

Telehealth is clearly a growing intervention area, particularly as a means of supporting people with SCI post-discharge who live in rural settings. This presents opportunities for countries with people dispersed over large rural and remote area such as the case in Australia, Canada, Brazil, China and India. It has been reported that people living in rural and remote areas have poorer access to primary care and face more inequity in health outcomes than people residing in major towns and cities [23] and therefore, research utilising telehealth has merit for informing future service delivery models and measuring health outcomes and other benefits for people with limited access to resources.

The lack of high-quality evidence restricts the profession’s ability to question current practices or optimise patient outcomes. This is not exclusive to SCI nursing, it is a broader issue across healthcare where it has been reported that up to 40% of patient care is unsupported by best evidence [3].

### Implications for nursing leadership and research capacity

The limited nurse authorship in many of the RCTs highlights the risk that nurses’ perspectives are underrepresented in SCI research agendas. This likely reflects two key factors: a lack of recognition by research teams of the unique contributions nurses can make to the research process, and limited access to the skills, resources, and funding necessary for nurses to lead research initiatives. Our review demonstrated that only 30% of the RCTs identified nurses as first or last author. The lack of nursing research leadership is a missed opportunity because nurses, by virtue of their proximity to patients and deep understanding of the lived experience of SCI, are uniquely positioned to identify clinically relevant research questions and to develop interventions that reflect patient priorities/relevance. Being a part of the research community helps shape the healthcare system and ultimately, patient outcomes [5], therefore nurses need to be involved in all phases of the research process to gain confidence and they need to be leading research [24].

The findings of this scoping review underscore an urgent need to strengthen nursing leadership in SCI research. Melnyk and Fineout-Overholt [25] argue that substantial education and training resources in research are essential to acquire the skills and confidence to implement best practice in the clinical setting. However, structural barriers, heavy workloads, limited research training, and inadequate institutional support continue to constrain the capacity of nurses to lead and publish high-quality RCTs [26, 27]. Systemic reform is imperative to address these barriers. Protected research time, mentorship programmes, and targeted funding for nurse-led SCI research would help to rebalance authorship representation and foster a stronger culture of inquiry. Collaboration between clinicians and academic partners could also accelerate capacity-building and methodological rigour.

### Methodological concerns

Most of the identified RCTs were vulnerable to bias with a median (IQR) PEDro score of 5.5 (5 to 7). A common limitation across these RCTs was inadequate blinding of participants, therapist and assessors. Some others were the assessment of the primary outcome in fewer than 85% of participants, and failure to use intention-to-treat analyses. These trial design linitations weaken our confidence in the results. The authors acknowledge that it is almost always impossible to blind participants and the clinicians administering the intervention so these items on PEDro are more a reflection on the vulnerability to bias rather than methodological quality. However, it is always possible to blind assessors.

The frequent failure to conceal allocation is particularly problematic, as it increases the risk of selection bias which can inflate the size of treatment effects. Although some high-quality studies (scoring 8–9) were identified, more needs to be done to ensure that all RCTs are of high quality. More also needs to be done to ensure that nurse researchers understand the importance of key methodological issues important for minimising bias [28, 29].

Several RCTs originated from LMICs, where there are often considerable variations in the scope-of-practice of nurses. In some cases, the interventions were not provided by nurses. These included interventions for wound care, health education, or ongoing clinical support. Instead, allied health professionals, technicians, or health workers were used. This may reflect workforce shortages and resource constraints. While such task-shifting can expand access to care for patients, particularly in rural or under-resourced settings, it risks diminishing the nursing contribution to SCI care and limiting the delivery of nursing-specific, evidence-based interventions. Therefore, any move toward task-shifting must be accompanied by clear frameworks that preserve the integrity of nursing practice and safeguard the delivery of high-quality, evidence-based SCI care.

### Limitations

This review has several limitations. Firstly, it only include RCTs published in English and did not include a search of grey literature. This may have resulted in the omission of relevant RCTs which introduces publication and language bias. However, scoping reviews are intended to map the breadth and characteristics of an evidence base rather than provide a fully exhaustive synthesis. Restricting the review to English and indexed databases enabled a feasible and reproducible search strategy within the available resources.

The review was limited to three databases and studies published between 2004 and 2024, potentially excluding older or unpublished RCTs. The authors chose MEDLINE, Embase and CENTRAL because they are considered to be the major databases for identifying RCTs and evidence relevant to healthcare interventions. The decision to limit the search to these databases reflected a pragmatic balance between comprehensiveness and feasibility, consistent with the exploratory aims of a scoping review. Although unpublished RCTs may contribute to publication bias, peer-reviewed RCTs indexed in major biomedical databases are most likely to influence evidence-based SCI nursing practice, clinical guidelines and future research directions. Therefore, the review focused on published literature to provide an overview of the accessible evidence underpinning current practice.

The 20-year timeframe was chosen to provide an overview of contemporary evidence underpinning SCI nursing interventions. Clinical practice, assistive technologies, telehealth delivery models and the role of nurses in SCI care have evolved considerably over the past two decades; therefore, older studies were considered less likely to reflect current practice contexts.

### Future directions

Through this scoping review process, we have identified opportunities for nurses and people with lived experience to be more involved in research. We will be designing a research project looking at prioritising research questions that are important to both nurses and people living with SCI. The aim will be to develop questions that can be investigated using high quality RCTs. The standard format for organising RCT questions, is by identifying the population, intervention, control and outcome and is generally referred as “PICO”.

## Conclusion

Our scoping review demonstrated that few RCTs have been conducted on nursing related interventions over the past twenty years. Of those that have been conducted, only one third were nurse led as indicated by a nurse as first or last author, and overall the RCTs were vulnerable to selection bias. This review highlights the need for greater nurse involvement and leadership in the research process to design and conduct high-quality RCTs that address clinical questions meaningful to people living with SCI, and to ensure that nurses can translate the findings into practice to achieve positive patient outcomes.

## Supplementary information

Supplementary File 1.

Supplementary File 2.

## Data Availability

All available individual data are provided in the paper.
