# Supplementary material for: Randomised controlled trials of nursing interventions in spinal cord injury: a scoping review
Source: Spinal Cord Ser Cases. 2026 Sep 3;12:22. doi: 10.1038/s41394-026-00749-x (PMC13542250; doi:10.1038/s41394-026-00749-x)
Supplement: Supplementary file 2 [file 41394_2026_749_MOESM2_ESM.docx]

**Supplementary File 2.	Search Terms**
